# Co-Detection of EBV and Human Polyomavirus JCPyV in a Case of AIDS-Related Multifocal Primary Central Nervous System Diffuse Large B-Cell Lymphoma

**DOI:** 10.3390/v15030755

**Published:** 2023-03-15

**Authors:** Mallory T. Barbier, Luis Del Valle

**Affiliations:** 1 Louisiana Cancer Research Center, Louisiana State University Health Sciences Center, New Orleans, LA 70112, USA; 2 Department of Pathology, Louisiana State University School of Medicine, New Orleans, LA 70112, USA

**Keywords:** diffuse large B-cell lymphoma, primary CNS lymphoma, Epstein–Barr virus, JCPyV, T-antigen, LMP, EBNA1, immunohistochemistry

## Abstract

The human neurotropic *Polyomavirus* JCPyV is the widespread opportunistic causative pathogen of the fatal demyelinating disease progressive multifocal leukoencephalopathy; however, it has also been implicated in the oncogenesis of several types of cancers. It causes brain tumors when intracerebrally inoculated into rodents, and genomic sequences of different strains and expression of the viral protein large T-Antigen have been detected in a wide variety of glial brain tumors and CNS lymphomas. Here, we present a case of an AIDS-related multifocal primary CNS lymphoma in which JCPyV genomic sequences of the three regions of JCPyV and expression of T-Antigen were detected by PCR and immunohistochemistry, respectively. No capsid proteins were detected, ruling out active JCPyV replication. Sequencing of the control region revealed that Mad-4 was the strain of JCPyV present in tumor cells. In addition, expression of viral proteins LMP and EBNA-1 from another ubiquitous oncogenic virus, Epstein–Barr, was also detected in the same lymphocytic neoplastic cells, co-localizing with JCPyV T-Antigen, suggesting a potential collaboration between these two viruses in the process of malignant transformation of B-lymphocytes, which are the site of latency and reactivation for both viruses.

## 1. Introduction

Primary central nervous system lymphoma (PCNSL) was first described in 1929 by Percival Bailey as perithelial sarcoma of the brain [1]. Over the next fifty years, there were several names to describe these primary CNS neoplasms, including perivascular sarcoma, microgliomatosis, malignant reticuloendotheliosis, and lymphoproliferative disorder. In 1974, James Henry clarified the varied terminology to be primary malignant lymphomas of the central nervous system [2]. Today, we use the terminology primary CNS lymphoma to describe rare, extranodal, malignant neoplasias of hematological origin restricted to the brain, spinal cord, leptomeninges, and ocular vitreoretinal compartment, without systemic involvement. The term “PCNSL” is frequently used synonymously to describe primary CNS diffuse large B-cell lymphoma (PCNS-DLCBL); however, in the fifth edition of the WHO Classification of Central Nervous System Tumours, the editors clarify the appropriate terminology stating that “Of the primary CNS lymphomas, diffuse large B-cell lymphoma of the CNS (CNS-DLBCL), previously called “primary CNS lymphoma” is the most common tumor type encountered” [3]. The distinction is important because while PCNS-DLBCL encompasses approximately 95% of PCNSLs, the remaining 5% includes Burkitt lymphoma, different low-grade B-cell lymphomas, and T-cell and NK/T-cell lymphomas, which have different and unique mechanisms of pathogenesis, varied immunophenotypes, and biological considerations. For this study, and in accordance with the WHO’s clarification, we use PCNS-DLBCL to specify a primary CNS non-Hodgkin’s lymphoma (NHL) subtype.

In immunocompetent individuals, PCNS-DLBCLs account for <1% of all non-Hodgkin lymphomas [4] and only 2.4% to 3% of all primary CNS tumors, with a median age of onset in the sixth to the seventh decade of life and a male-to-female ratio of 3:2 [3]. Immunocompetent PCNS-DLBCLs are typically associated with solitary brain lesions. On the other hand, immunocompromised PCNS-DLBCLs occur earlier, in the third to fourth decade of life, and are more likely to be multifocal brain lesions [5]. The lesions typically present as deep-seated masses, usually affecting the basal ganglia and cerebral hemispheres, and constitute one of the few entities that can affect the corpus callosum. These tumors can range from firm to soft and usually exhibit areas of necrosis and hemorrhage, particularly in immunocompromised individuals. On microscopic examination, these tumors start as perivascular cuffs of neoplastic lymphocytes that eventually break out of the Virchow–Robin space and diffusely infiltrate the brain parenchyma [6]. By immunohistochemistry, more than 90% of PCNS diffuse large-cell lymphomas are of B-cell origin, expressing CD20, and approximately only 2% are of T-cell origin [7,8].

Assorted studies describe two subtypes of DLBCLs using the Hans algorithm to predict the cell of origin [6,7,8,9]. This algorithm is loosely applied to DLBCLs of the CNS due to the variability of comorbidities and immunophenotypes associated with PCNS-DLBCLs. The more common subtype, germinal center B-cell-like (GCB), is characterized by CD10 positivity, MUM1/IRF4 negativity, and/or BCL6 positivity and has a 76% overall survival estimate [10]. The second subtype, nongerminal center B-cell-like (non-GCB), which represents an abnormal B-cell development stage, is characterized by CD10 negativity, BCL6 positivity, and MUM1/IRF4 positivity and has a worse prognosis with a 34% overall survival estimate. The non-GCB subtype corresponds with the activated B-cell-like (ABC) and not otherwise specified (NOS) subtypes.

PCNS-DLBCL development may be promoted by deregulation and errors in B-lymphocyte development [11,12]. Common genetic alterations in PCNS-DLBCLs include chromosomal translocations, with BCL6 and MYC among the most prevalent [12,13,14]. In 25% of cases related to the ABC subtype, BCL6 translocation is suggested to be the mechanism for preventing B-cells from completing the plasmacytic program [10,15,16]. Additionally, BCL6 translocation with non-Ig partners is associated with increased gene expression [17]. MYC is a cellular oncogene that encodes transcriptional regulators involved in controlling cell proliferation and death, and it has been shown that translocations increase MYC expression, which strongly promotes cell proliferation [18,19]. BCL6 and MYC translocations are described as double expressors when both are simultaneously expressed, and double expressors are associated with worse survival [20,21,22]. Aberrant somatic point mutations may also occur in PCNS-DLBCLs involving MYC and PAX5, also known as B-cell-specific activator protein (BSAP), which then become mutated oncogenes that contribute to the pathogenesis of lymphomas [10,23]. The nuclear transcription factor, MUM1/IRF4, is usually expressed in activated B- and T-cells and participates in B- and T-cell differentiation [24,25]. Immunohistochemistry for MUM1/IRF4 positivity in PCNS-DLBCLs is associated with the non-GCB subtype and indicates a lack of EZH2, an enzyme that suppresses the pro-plasmacytic program [11], and further indicates loss of function of the germinal center phenotype.

The human gammaherpesvirus, Epstein–Barr virus (EBV), discovered in 1964, is the well-established etiological pathogen linked to the development of PCNS-DLBCLs in immunodeficient individuals [26]. After primary infection, EBV remains latent in an estimated 90% of adults worldwide, intermittently becoming activated during instances of decreased immune activity but typically returning to a latent state once immune function stabilizes, with no apparent or long-term complications [27]. Latent EBV infections are characterized by the expression of a limited number of viral genes, including three latent membrane proteins, LMP1, LMP2, and LMP3 [28]. Several studies suggest that LMP1 plays a role in preventing apoptotic death via BCL-2 and involves the NF-κB pathway, leading to the preservation of EBV-infected B-cells for latency maintenance or viral replication [29,30]. An in vitro study demonstrated that EBV is capable of immortalizing primary B-cells into lymphoblastoid cell lines (LCLs), thus enabling B-cells to proliferate at a rate that doubles its population within 24 h [31]. LCLs abundantly express EBER1 and EBER2, are used for in situ detection, and have been implicated in interacting with cellular proteins that play a crucial role in antiviral innate immunity and modulation of innate immune signaling contributing to EBV-mediated oncogenesis [32]. PAX5 has been demonstrated to transactivate EBV via promoter regulation, indicating that EBV has evolved to use the B-cell-restricted nature of PAX5 [33]. EBV-related malignancies are usually positive for the B-cell markers CD20, CD19, PAX5, and CD79a and primarily negative for CD10; thus, CD10 positivity should prompt the search for systemic disease [34,35]. BCL6 mutations occasionally occur in EBV-positive PCNS-DLBCL [36,37].

Another pathogen that has been associated with primary CNS lymphomas is the human neurotropic JC virus (JCPyV), a member of the polyomavirus family that is the causative agent of progressive multifocal leukoencephalopathy (PML), a fatal demyelinating disease of the CNS [38]. JCPyV is also a ubiquitous infection among the worldwide human population, and it is speculated that primary infection with the archetype strain occurs during early childhood and persists for life by establishing latency in the kidney. Viral migration theories developed when JCPyV was detected in B-lymphocytes and mononuclear cells in brain perivascular spaces of PML patients, indicating the virus can establish latency in infected blood cells and migrate to other tissues [39,40]. In fact, JCPyV-infected cells and individual components of the virus have been detected in vitro and in vivo within various normal tissue types, such as the GI tract, tonsils, and brain [41,42,43].

JCPyV has a circular, closed double-stranded DNA genome, which can be divided into three regions, an early transcriptional region, in which transcription occurs before viral replication, and encodes for the functional proteins large T-Antigen and small t-antigen, a late transcriptional region, which is active after viral replication and contains genes for the capsid proteins VP1, VP2, and VP3, as well as the accessory product Agnoprotein, divided by the control regulatory region, which is responsible for the strain of JCPyV and contains the sites for activation [44]. Mutations in this control regulatory region are responsible for the different strains of JCPyV; the contagious strain that remains latent in kidney and lymphoid tissues is designated CY (archetype), while most mutations involve a duplication of the 98 base pairs, and these strains that have been found in PML and brain tumors. JCPyV-infected B-cells can undergo cellular transformation due to T-Antigen [40,45], which is a regulatory protein and designated oncoprotein implicated in tumorigenesis because of its ability to bind to and inactivate tumor suppressors p53 and pRb, dysregulate signaling pathways (Wnt and IGF), and interfere with faithful DNA repair. T-Ag inactivates pRb, which promotes cell cycle progression, while simultaneously binding to p53, preventing the p53 response to DNA damage [46,47,48].

Here, we present a rare case of a multifocal primary CNS lymphoma in a patient with AIDS, in which both EBV and JCPyV have been detected in tumor cells.

## 2. Materials and Methods

### 2.1. Case Report

A 28-year-old homeless, cachexic male with a past medical history of untreated, uncontrolled AIDS (CD4 count: 24 per cubic milliliter), multiple substance abuse, and drug-induced psychosis presented to the emergency department with psychomotor agitation, abnormal sensations, and generalized weakness. CT showed multiple heterogeneous masses in the left frontal, parietal, and left cerebellum with midline shift. The patient was admitted to the intensive care unit, where HART therapy and steroids were started. MRI showed a 5.4 × 4.9 cm mass in the right basal ganglia with midline shift, a second 3.3 × 2.3 cm mass in the left cerebral cortex, and a third 2 × 2 cm mass in the right cerebellar hemisphere. Neurosurgery conducted left frontal brain biopsy, which was diagnosed as a diffuse large B-cell lymphoma, and placed an extra ventricular drain to relieve the increased intracranial pressure. However, the patient’s mental condition deteriorated quickly, and he died of aspiration pneumonia and respiratory failure 23 days after admission.

### 2.2. Immunohistochemistry

After the autopsy, the brain tissues were fixed in 10% buffered formalin and embedded in paraffin. Sections of four microns in thickness were cut and placed on electromagnetically charged slides (Fisher Scientific, Fair Lawn, NJ, USA) to prevent detachment. Hematoxylin and Eosin staining was performed for routine histopathological examination. Tumors were diagnosed according to the latest World Health Organization Classification of Tumors of the Nervous System. Immunohistochemistry was performed using the avidin–biotin–peroxidase methodology, according to the manufacturer’s instructions (Vectastain ABC Elite Kit, Vector Laboratories, Burlingame, CA, USA). Our modified protocol included deparaffination in xylenes, rehydration through descending grades of ethanol up to water, nonenzymatic antigen retrieval with 0.01 M sodium citrate buffer pH 6.0 at 95 °C for 25 min, endogenous peroxidase quenching with 3% H_2_O_2_ in methanol, blocking with normal horse serum (for mouse monoclonal antibodies) or normal goat serum (for rabbit polyclonal or recombinant rabbit monoclonal antibodies), and incubation with primary antibodies overnight at room temperature in a humidified chamber. Antibodies for viral proteins included mouse monoclonals anti-SV40 T-Antigen, which cross-reacts with the T-Antigen of JCPyV (clone PAb416, 1:100 dilution, Millipore/Sigma, Burlington, MA, USA), EBV nuclear antigen/EBNA1 antibody (clone E1-2.5, 1:100 dilution, Abcam, Cambridge, MA, USA), EBV, LMP (clone CS.1-4, 1:50 dilution, DAKO/Agilent, Santa Clara, CA, USA); p53 was detected with a mouse monoclonal antibody (clone DO-7, 1:50 dilution, DAKO/Agilent, Santa Clara, CA, USA); antibodies for cellular markers included mouse monoclonals against CD20 (clone L26, 1:100 dilution, DAKO/Agilent, Santa Clara, CA, USA), Bcl-2 (clone C-2, 1:100 dilution, Santa Cruz Biotechnology, Dallas, TX, USA), Bcl-6 (clone SPM602, 1:100 dilution, Abcam, Cambridge, MA, USA), c-MYC (clone 9E10, 1:100 dilution, Santa Cruz Biotechnology, Dallas, TX, USA), a rabbit monoclonal against MUM1/IRF4 (clone SP114, 1:100 dilution, Abcam, Cambridge, MA, USA), and a rat monoclonal against PAX5 (clone 1H9, 1:100 dilution, Abcam, Cambridge, MA, USA). After rinsing in PBS, sections were incubated with biotinylated secondary antibodies for 1 h, followed by incubation with avidin–biotin–peroxidase complexes for 1 h, both at room temperature in a humidified chamber. Finally, the peroxidase was developed with diaminobenzidine (Boehringer, Mannheim, Germany) for 3 min, and the sections were counterstained with hematoxylin and mounted with Permount (Fisher Scientific). Photomicrographs were taken with an Olympus DP72 Digital Camera using an Olympus BX70 microscope (Olympus, Center Valley, PA, USA).

### 2.3. Double-Labeling Immunofluorescence

The first part of our protocol for double-labeling was similar to the methodology described above, with the exception of the endogenous peroxidase quenching step, which was omitted as the antibodies would be fluorescently tagged. After incubation with the first primary antibody (T-Antigen, mouse monoclonal), an Alexa Flour 488-conjugated anti-mouse secondary antibody was incubated for 1 h in the dark. Sections were then washed thoroughly with PBS, blocked again, and a second primary antibody raised in a different species than the first one (LMP, rabbit monoclonal) was incubated overnight. Finally, a second Alexa Fluor 568-conjugated anti-rabbit secondary antibody was incubated for 1 h in the dark, and slides were cover-slipped with an aqueous mounting media containing DAPI (Vectashield Plus Antifade, Vector Laboratories) and visualized in an Olympus FV100 confocal microscope.

### 2.4. DNA Extraction and PCR Amplification

DNA extraction, PCR amplification, and Southern blot hybridization were performed as described previously [49]. DNA was extracted from four sections of 10 μm each, from an area of the tumor containing exclusively neoplastic cells using the QIAamp tissue kit (Qiagen, Valencia, CA, USA). PCR amplification was performed on the extracted DNA with four individual sets of primers: Pep1 and Pep2, which amplify sequences in the N-terminal region of the JCPyV T-Antigen (nucleotides 4255 to 4274 and 4408 to 4427, respectively); CR2 and CR3, which amplify the control regulatory region of JCPyV (nucleotides 238 to 257 and 5101 to 5121, respectively); VP2 and VP3, which amplify regions of the JCPyV VP1 capsid protein (nucleotides 1828 to 1848 and 2019 to 2039, respectively); Agno1 and Agno2, which amplify sequences within the coding region of the JCPyV Agnogene (nucleotides 279 to 298 and 438 to 458, respectively). Amplification was carried out on 500 ng of template DNA with Failsafe *Taq* polymerase in Failsafe Buffer B in a total volume of 50 μL containing 0.5 nM of the primers. After denaturation at 95 °C for 10 min, 45 cycles of denaturation at 95 °C for 15 s, annealing for 30 s, and extension at 72 °C for 30 s, a final extension step of 72 °C for 7 min was performed for termination. Annealing temperatures were 55 °C for Pep primers, 54 °C for VP primers, 57 °C for Agno primers, and 55 °C for CR primers. In parallel, 500 ng of plasmid DNA containing JCPyV, SV40, or BKV sequences was amplified to serve as positive and negative controls. Southern blot analysis was performed using 15 μL of each of the PCR products separated by 2% agarose gel electrophoresis, depurated, denatured, and transferred from the gel into nylon membranes (Hybond-N; Amersham). The membranes were hybridized with 10^6^ cpm of γ-^32^P-end-labeled oligonucleotide probes/mL overnight at 65 °C. To remove nonspecific binding, blots were washed twice in 2X SCC/0.1% SDS at 55 °C for 5 min, followed by washing and autoradiography. Oligonucleotides homologous to the following JCV-specific sequences were utilized as probes: T-Antigen probe (Pep primers; nucleotides 4303 to 4327), VP probe (nucleotides 1872 to 1891), and Agno probe (nucleotides 425 to 445), as well as CR probe (nucleotides 68 to 81). After extraction of the amplified fragments, sequencing of the CR was performed using the Applied Biosystems Prism 377 DNA sequencer XL.

## 3. Results

### 3.1. Gross and Histopathological Aspects of the Tumor

**Figure 1:**
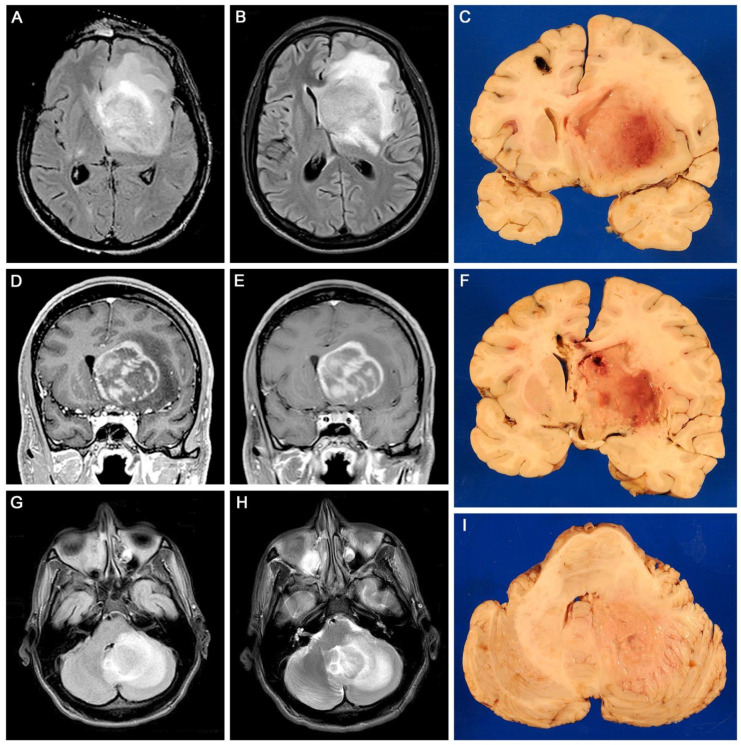
Neuroimaging and neuropathology: Axial plane MRI shows a large mass in the right basal ganglia which causes significant edema (Panels (**A**,**B**)). Coronal MRI shows the mas is ring enhancing (Panels (**D**,**E**)). Coronal sections of the brain corroborated a large mass of poorly defined edges with prominent areas of necrosis, located in the right basal ganglia with areas of necrosis, that causes significant edema and compresses the lateral ventricle; the site of the surgical probe can be seen on the contralateral frontal lobe (Panels (**A**–**F**)). Another similar homogeneous mass can be seen in the right cerebellar hemisphere, causing a prominent midline shift (Panels (**G**–**I**)).

Gross examination of coronal sections of the brain revealed a large mass of irregular, poorly defined edges located in the right basal ganglia, which measured 5 × 5 × 4 cm, and extended rostrally and medially into the corpus callosum and caudally into the parietal lobe. This mass was homogeneous, grey, and of soft, granular consistency. It caused significant edema, which produced the partial compression of the lateral ventricle and subfalcine herniation of the cingulate gyrus. A second lesion of similar characteristics was found in the left frontal lobe, measuring 3 × 2 cm, in which the tract of the surgically implanted drain was found. A third mass, also homogeneous, grey and soft, and of poorly defined edges, measured 3 × 2.5 cm and was found in the right cerebellar hemisphere. Figure 1 depicts the neuroimaging and gross aspects of the tumors.

**Figure 2:**
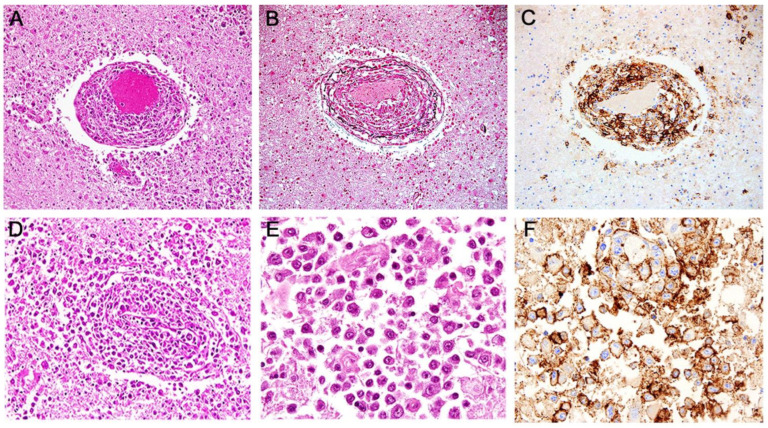
Histological and immunohistochemical profile of the tumor: Microscopically, the tumor is composed by numerous sheets of neoplastic cells, which in the periphery of the tumor are confined to the Virchow–Robin space (Panel (**A**), H&E), layered within reticulin fibers (Panel (**B**), reticulin), and in more central areas, break and invade into the brain parenchyma (Panel (**D**), H&E). At higher magnification, the neoplastic cells are round, with some degree of nuclear pleomorphism and scant cytoplasm (Panel (**E**), H&E). Immunohistochemically, the neoplastic cells express CD-20 (Panel (**C**)), and CD-79b (Panel (**F**)), confirming their B-cell phenotype.

Histologically, the three tumors were composed by numerous sheets of atypical and pleomorphic lymphoid cells with increased mitotic activity. In the periphery of the tumor, these neoplastic lymphocytes were confined to the Virchow–Robin space, as perivascular cuffs (Figure 2A,B), and in more central areas, they broke out into the brain parenchyma and formed homogeneous sheets of tumors cells (Figure 2D,E).

### 3.2. Immunohistochemical Characterization of the Tumor

Tumor cells were robustly immunoreactive to CD-20 and CD-79b, corroborating their B-cell phenotype (Figure 2C,F, respectively). As expected, since more than 90% of primary CNS lymphomas are of B-cell origin, CD3 expression was negative. Next, in order to establish the subtype of lymphoma, we performed immunohistochemistry for PAX5, a transcription factor indicative of lymphoblastic cells, which showed robust nuclear labeling, and MUM1/IRF4, another transcription factor that identifies nongerminal center B-cells and that has been linked with poor survival and indicates a lack of EZH2, an enzyme that suppresses the pro-plasmacytic program, and further indicates loss of function of the germinal center phenotype. MUM1/IRF4 was also strongly positive in tumor cells, and considered together with PAX5 positivity, indicated the nongerminal center origin of this tumor. CD5 negativity ruled out a small cell mantle lymphoma, and CD10 negativity ruled out a follicle center lymphoma. These results are important because they suggest that the malignant transformation process occurred somewhere other than in the lymphoid organs, most likely in circulating blood, where two important pathogens, EBV and JCPyV, are known to cause chromosomal alterations and dysregulate oncogenic pathways in lymphocytes.

### 3.3. Expression of EBV and JCPyV Viral Proteins

**Figure 3:**
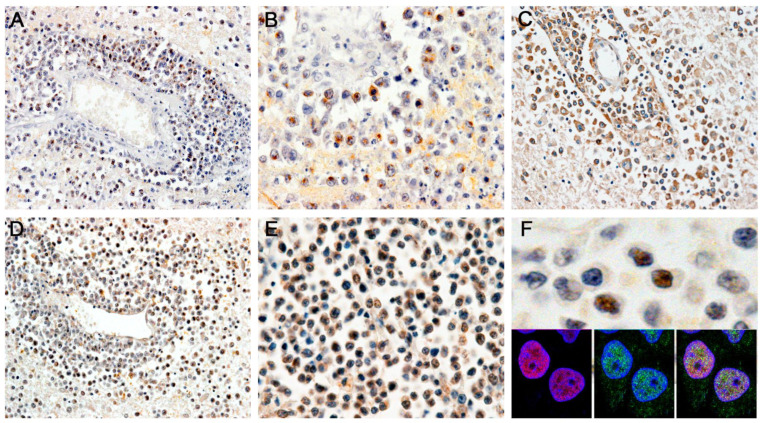
Immunohistochemical detection of EBV and JCPyV proteins: Immunohistochemistry for Epstein–Barr viral proteins showed the presence of the latent viral protein LMP1 in perivascular lymphocytic tumor cells (Panels (**A**,**B**), 100 and 400× magnification, respectively), and the EBER1 in the cytoplasm of the same cuffs of neoplastic lymphocytes (Panel (**C**), original magnification 100×). Large T-Antigen of the human neurotropic JCPyV was robustly expressed in the nuclei of several, but not all, perivascular neoplastic lymphocytes (Panels (**D**–**F**), original magnification 100×, 400×, and 1200×, respectively). Double-labeling demonstrated the co-localization of both T-Antigen and EBNA1 in the nuclei of neoplastic cells, Panel (**F**), inserts).

As discussed in the introduction, the Epstein–Barr virus is the known pathogen linked to primary CNS lymphomas. To establish the presence of EBV in tumor cells, we performed immunohistochemistry with two specific antibodies. The nuclear antigen (EBNA1), whose functions include the replication and segregation of EBV episomes, promotion of lytic infection, and the activation of latent genes important for cell immortalization, was found robustly expressed in most neoplastic lymphocytes (Figure 3A,B). The latent membrane protein (LMP), which when activated is crucial for malignant transformation, was also expressed in the cytoplasm of virtually all tumor cells, both in the Virchow–Robin space and infiltrating the brain parenchyma cells (Figure 3C). Next, to establish the presence of JCPyV active transcription, we performed immunohistochemistry with an antibody for SV40 T-Antigen, which cross-reacts with the T-Antigen of JCPyV, and found expression of the oncogenic protein in the nuclei of the majority, but not all, tumor cells, pointing to the “hit and run” mechanism theory, in which T-Antigen is required for malignant transformation, but once that event has happened, its expression is slowly extinguished (Figure 3D–F). Furthermore, double-labeling immunofluorescence demonstrated the co-localization of both T-Antigen and EBNA-1 in the nuclei of neoplastic lymphocytes (Figure 3 insert), indicating the presence of both viruses and their products within the same cellular compartment and suggesting a possible crosstalk between the two oncogenic viruses.

### 3.4. Detection of JCPyV Genomic Sequences

**Figure 4:**
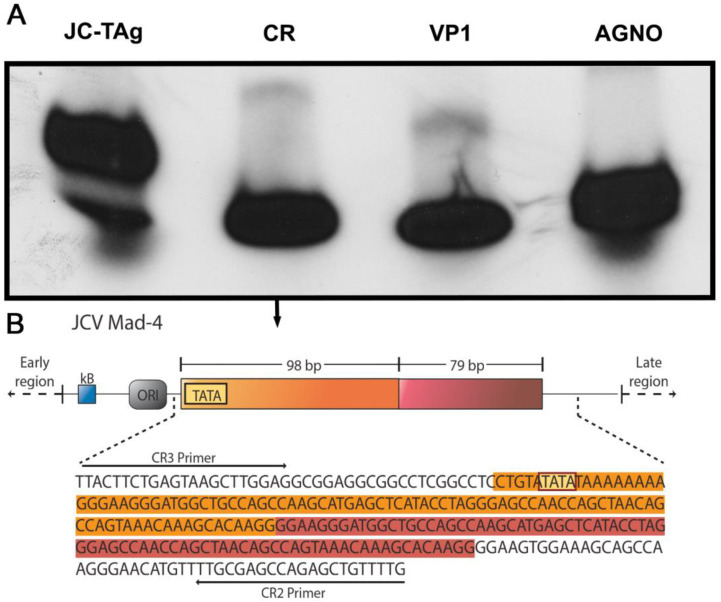
PCR Amplification of JCPyV genomic sequences: DNA extraction and PCR amplification from an area of tumor cells showed the presence of genomic sequences from all regions of JCPyV (Southern blot, Panel (**A**)). Furthermore, sequencing of the control region amplified sequence revealed that the strain present was Mad-4. A schematic representation of the CR is shown along with the sequence and location of the primers used Panel (**B**)).

In order to corroborate the presence of JCPyV genomic sequences in the tumor cells, we performed PCR amplification and Southern blot hybridization in a section that exclusively contained tumor cells, with specific primers and probes for the three transcriptional genes of JCPyV, the carboxy-terminal of the T-Antigen gene, the Agnogene, and the capsid-encoding gene VP1, and found that all regions of the virus were present in the tumor cells. Furthermore, the control regulatory region was also present (Figure 4A). We then proceeded to sequence the amplified segment of the control region, finding that Mad-4 was the strain of JCPyV present in the tumor cells in this case (Figure 4B).

## 4. Discussion

The mechanisms and pathogenesis for PCNS-DLBCLs are still largely unknown. Immunodeficiency is the only known risk factor for developing DLBCLs of the CNS, which is the most common type of lymphoma reported in AIDS patients. HIV-induced immunodeficiency has been extensively associated with lymphomagenesis [10,50,51], and is also a significant risk factor for the acquisition of opportunistic viral infections such as EBV and JCPyV [35,52,53]. How viruses, in this case HIV, EBV, and JCPyV, interact with each other and the host to stimulate oncogenesis is still a popular topic of debate. Given these viruses’ oncogenic potential, we analyzed an interesting case of AIDS-related PCNS-DLBCL that exhibited both EBV and JCPyV viral components in neoplastic cells.

Many studies have associated chromosomal alterations with AIDS-related PCNS-DLBCLs [11,12,13,23,54,55,56,57,58,59], and a handful of viruses have been confirmed to induce the chromosomal alterations needed for tumorigenesis, including EBV and JCPyV. EBV-positive DLBCLs are associated with a worse overall survival [60,61,62], but these data have yet to be elucidated in regards to JCPyV. Previous studies have demonstrated the presence of EBV and JCPyV in B-lymphocytes [40,63,64,65]. Since EBV is a highly prevalent virus that latently infects people worldwide, the incidence rates for CNS lymphomas would be much higher if EBV were able to induce oncogenesis on its own. We know EBV can infect B-cells and has been shown to be present in 70–80% of EBV-positive AIDS-related DLBCLs. This suggests that EBV plays a role in HIV-related lymphomagenesis [35,66,67]. In addition, the latent membrane protein 1 (LMP1) of EBV is essential for EBV-immortalized B-cell proliferation [68]. However, since PCNSLs are extremely rare and account for only 2–3% of all brain tumors [60,69], EBV infection most likely acts as a prerequisite that must then be stimulated by some other type of process, for instance, infectious, immune, or inflammatory, to achieve oncogenesis. CNS coinfection with JCPyV and EBV has been documented in AIDS patients, demonstrating there are reported co-occurrences of these two opportunistic infections together in immunocompromised individuals [53]. There have been multiple reports of PML cases with concomitant PCNSLs, implicating JCPyV as a potential activator of EBV and a contributor in the development of CNS malignancies [70,71,72,73]. In addition, large studies have found the presence of JCPyV genomic sequences and expression of viral proteins in cases of PCNSLs, in which tumors with coactivation of both JCPyV and EBV exhibited a higher frequency of chromosomal aberrations and rearrangements when compared with only JCPyV activation [46,74]. Regarding the development of PCNSLs, it has been suggested that JCPyV may act as a co-factor or induce additional “hits” that allow for B-cell immortalization and transformation in some CNS lymphomas [46,74]. In healthy individuals, persistent EBV infections are tightly regulated by T-lymphocytes to maintain an asymptomatic status, but in the context of AIDS, dramatic decreases in T-cells support uncontrolled viral proliferation.

JCPyV is another ubiquitous human virus that has been detected in approximately 70% of the worldwide population and is able to infect various cell types, including B-lymphocytes and mononuclear cells [75], in which the virus remains in a latent state [76,77]. The virus was also detected in various types of tumor cells of CNS origin, including PCNSLs [48]. Many studies have demonstrated that JCPyV transformation of nonpermissive cells, or cells that do not support JCPyV replication, is a result of JCPyV-induced genomic instability. This instability may be introduced when latent JCPyV-infected B-lymphocytes leave reservoir sites such as the tonsils or kidneys and travel through the blood stream, where rearrangement of the noncoding control region (NCCR) occurs, or when infected cells enter germinal centers. JCPyV’s ability to transform B-cells was demonstrated both in vivo and in vitro by interacting with p53 and pRb proteins, and cell cycle regulators, resulting in “rogue cells.” “Rogue cells” are cells that contain multiple unstable chromosomal aberrations, including aneuploidy mitoses [45,46,65,75]. JCPyV infection of glial cells has been associated with DNA damage and chromosomal aberrations [45], demonstrating that JCPyV infection and more specifically, T-Ag expression results in a large induction of the expression of Rad51, an HR-DNA repair protein. When Rad51 is overly expressed, alternative double-strand break (DSB) repair pathways are promoted, resulting in chromosomal breaks, genomic instability, and aneuploidy [78]. T-Ag can also bind directly to β-catenin and induce its translocation to the nucleus, subsequently enhancing c-MYC, which is a powerful proto-oncogene involved in cell cycle control, DNA and energy metabolism, and apoptosis [19,42,48]. When β-catenin is bound to IRS-1 in the nucleus, or insulin receptor substrate-1, which is a part of the IGF-1R pathway, Rad51 is inactivated, forcing the cell to repair DSBs using nonhomologous end joining (NHEJ) that results in translocations and telomere fusion [79,80]. These are hallmarks of tumor cells. IGF-1R and T-Ag can also reinitiate survivin production, preventing apoptosis of infected cells [38,81]. It has also been demonstrated that high titers of JCPyV antibodies correlate with an increased frequency of chromosomal aberrations in human lymphocytes [82]. Indeed, lymphoid precursor cells are well-suited sites of JCPyV NCCR alterations due to their specialized DNA recombination apparatus and how they carry out extensive DNA recombination and DNA replication in the blood [83,84]. Specific NCCR DNA sequences can form palindrome secondary structures that stall replication elongation, resulting in double-stranded DNA breaks [85]. These DSBs are usually repaired by recombination mechanisms, but as previously stated, in the presence of JCPyV’s T-Ag, the recombination mechanisms are altered and result in genome deletions and duplications [83].

**Figure 5:**
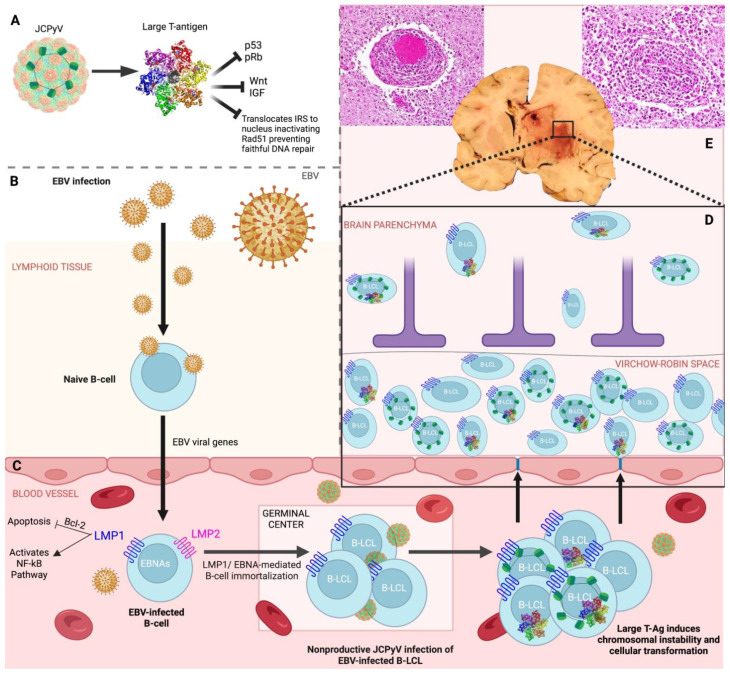
Model of EBV and JCPyV oncogenesis in primary CNS lymphomas: (**A**) JCPyV encodes for large T-Antigen (T-Ag) that inactivates tumor suppressors, p53 and pRb, dysregulates signaling pathways, Wnt and IGF, and prevents faithful DNA repair by initiating IRS translocation to the nucleus, inactivating Rad51. (**B**) EBV viral gene integration secondary to infection of naive B-cells during viral latent state. (**C**) EBV-infected B-cells express viral proteins, LMP1, LMP2, and EBNAs. LMP1 inhibits apoptosis via Bcl-2 and activates the NF-κB pathway. EBV viral proteins LMP1 and EBNA mediate B-cell immortalization. JCPyV nonproductively infects EBV-immortalized B-lymphoblastoid cell line (B-LCL) and translates T-Ag that induces chromosomal instability, resulting in DNA-damaged, malignant B-cells that cross the blood brain barrier. (**D**) Pleomorphic, neoplastic B-cells proliferate in the Virchow–Robin space and infiltrate brain parenchyma. (**E**) Neoplastic cells form perivascular cuffs that break out of the Virchow–Robin space, resulting in an irregular, necrotic lesion in the basal ganglia.

One model suggests that in an immunosuppressed environment, viruses can evade the immune system by inducing a latent infectious state that promotes a damaged and decreased T-cell response that is unable to clear out infected cells, while simultaneously stimulating an overactivated, yet impaired B-cell response that is unable to effectively identify and present antigens to the dysfunctional T-cells. The infected cells accumulate and utilize both their own viral proteins and the host’s cellular machinery to deregulate host cellular pathways. One study confirmed a high prevalence of JCPyV DNA in circulating B-lymphocytes [63], and another study observed JCPyV DNA in the plasma of AIDS patients, which supports the theory of peripheral virus reservoirs, and that AIDS-related immunosuppression may reactivate JCPyV latency, increasing the probability of mutations [64]. JCPyV was also more frequently detected in HIV-positive bone-marrow samples, suggesting that HIV-positive patients may experience an extended and more severe immune suppression that promotes JCPyV reactivation. The bone marrow compartment is an ideal site for long-term viral latency, further suggesting hematogenous dissemination as a potential mechanism to gain entry to the CNS [86]. In fact, one group observed that JCPyV nonproductively infects EBV-transformed B-cells that may act as a potential vehicle by which JCPyV can cross the blood brain barrier [63]. Figure 5 shows our proposed model of EBV and JCPyV oncogenesis in CNS lymphomas in which T-Antigen acts as an important co-factor that causes DNA damage, and alterations in faithful DNA repair mechanisms.

## Data Availability

Not applicable.
